# Procalcitonin at 12–36 hours of fever for prediction of invasive bacterial infections in hospitalized febrile neonates

**DOI:** 10.3389/fped.2022.968207

**Published:** 2022-09-29

**Authors:** Anne-Sophie Romain, Romain Guedj, Anais Chosidow, Nicolas Mediamolle, Aurélie Schnuriger, Sophie Vimont, Charlène Ferrandiz, Nicolas Robin, Marie-Hélène Odièvre, Emmanuel Grimprel, Mathie Lorrot

**Affiliations:** ^1^Department of General Pediatrics, Trousseau Hospital, Assistance Publique-Hôpitaux de Paris (AP-HP), Sorbonne Université, Paris, France; ^2^Department of Pediatrics Emergency, Trousseau Hospital, Assistance Publique-Hôpitaux de Paris (AP-HP), Sorbonne Université, Paris, France; ^3^Perinatal and Pediatric Epidemiology Research Team (EPOPé), Centre de Recherche Épidémiologie et Statistique Sorbonne Paris Cité (CRESS) INSERM UMR1153, Paris, France; ^4^Department of Virology, Trousseau Hospital, Assistance Publique-Hôpitaux de Paris (AP-HP), Sorbonne Université, Paris, France; ^5^Sorbonne Université, INSERM UMR_S 938, Centre de Recherche Saint Antoine (CRSA), Paris, France; ^6^Department of Bacteriology, Saint Antoine Hospital, Assistance Publique-Hôpitaux de Paris (AP-HP), Sorbonne Université, Paris, France; ^7^INSERM UMR_S 1155, Hôpital Tenon, Paris, France; ^8^Biologie intégrée du globule rouge, UMR_S1134, INSERM, Université de Paris, Paris, France

**Keywords:** neonates, procalcitonin, invasive bacterial infection, fever, antibiotics

## Abstract

**Aim:**

We aimed to investigate the performance of procalcitonin (PCT) assay between 12 and 36 h after onset of fever (PCT H12-H36) to predict invasive bacterial infection (IBI) (ie, meningitis and/or bacteremia) in febrile neonates.

**Methods:**

We retrospectively included all febrile neonates hospitalized in the general pediatric department in a teaching hospital from January 2013 to December 2019. PCT assay ≤ 0.6 ng/ml was defined as negative. The primary outcome was to study the performance of PCT H12-H36 to predict IBI.

**Results:**

Out of 385 included neonates, IBI was ascertainable for 357 neonates (92.7%). We found 16 IBI: 3 meningitis and 13 bacteremia. Sensitivity and specificity of PCT H12-H36 in the identification of IBI were, respectively, 100% [95% CI 82.9–100%] and 71.8% [95% CI 66.8–76.6%], with positive and negative predictive values of 14.3% [95% CI 8.4–22.2%] and 100% [95% CI 98.8–100%] respectively. Of the 259 neonates who had a PCT assay within the first 12 h of fever (< H12) and a PCT assay after H12-H36, 8 had IBI. Two of these 8 neonates had a negative < H12 PCT but a positive H12-H36 PCT.

**Conclusions:**

PCT H12-H36 did not miss any IBI whereas < H12 PCT could missed IBI diagnoses. PCT H12-H36 might be included in clinical decision rule to help physicians to stop early antibiotics in febrile neonates.

## Introduction

About 5 to 15% of febrile infants under 29 days of age evaluated in the emergency department (ED) have invasive bacterial infection (IBI) defined as meningitis or bacteremia and severe bacterial infection (SBI) without bacteremia including mainly urinary tract infection (UTI) (1–4). Whereas urinalysis is highly sensitive for detecting UTI in febrile neonates (5), actual ED clinical prediction rules combining clinical and biological data (6–8) fail to recognize all IBI in febrile neonates (9). Because missed-IBI may lead to severe complications (10), all febrile neonates are classified as at high risk of IBI regardless of any clinical or biological factors, and their treatment involves broad-spectrum antibiotic intravenous administration and hospitalization (11).

Excessive antibiotic use and prolonged length of stay (LOS) in this population can have unintended consequences such as iatrogenic complications, disruption in breastfeeding, parental psychological stress and resource overutilization with high cost (12). However, evidence is lacking to help physicians to decrease the LOS and of antibiotic administration once a febrile neonate is hospitalized. Clinical appearance alone may not be sufficient (9). Virus detection makes the risk of IBI lower but non-negligible (13). Finally, recent studies have showed that growth of bacteria in blood culture is detectable in about 90% and 95% of the samples within 24 h and 36 h respectively. As a consequence, LOS and antibiotic management have been showed highly variable in pediatrics units with a minimum 48 h waiting period which corresponds to the culture time of the bacteriological samples (14).

Several studies have evaluated the impact of the use of procalcitonin (PCT) for diagnosis and to guide therapeutic decision making with consequent reduction in the duration of antibiotic therapy in patients with pneumonia or hospitalized in intensive care unit (15–17). PCT has largely increased the performance of ED clinical prediction rules in detecting IBI in neonates, even though missing a bacteremia remains possible (18), but no studies have evaluated its performance in febrile neonates hospitalized. PCT can be detected in the plasma 2–3 h after an injection of endotoxin and peaks around 12 h after the onset of infection (19, 20).

We therefore hypothesized that neonates with IBI would all have a positive PCT between 12 and 36 h after the onset of the fever (PCT H12-H36) since PCT could be negative when tested too early after the onset of fever.

Our primary objective was to study the performance of PCT H12-H36 to predict IBI. The secondary objectives were to study the performance of PCT H12-H36 to predict SBI and to compare the performance of the PCT within the first 12 h of the onset of the fever vs PCT H12-H36 to detect IBI and SBI.

## Methods

### Study design and setting

We retrospectively reviewed the records of all febrile neonates hospitalized in the general pediatric department of one pediatric teaching hospital from January 2013 to December 2019 in Paris, France. In our pediatric ED, guidelines for all febrile neonates direct the physician to obtain blood, urine and cerebral spinal fluid (CSF) cultures, undertake C reactive protein (CRP) and PCT assays, deliver broad spectrum antibiotics and admit in a hospitalization unit for observation. Our pediatric unit guidelines also recommend performing a PCT H12-H36 in order to help in the decision to stop administering the antibiotics. We determined the proportions of IBI and SBI in our population and evaluated the performance of the PCT H12-H36 to predict them.

### Population selection

Neonates aged 4 to 28 days hospitalized in the general pediatric department for fever (temperature ≥ 38°C reported by the parents or notified in the ED) were included regardless the presence or not of an obvious source of the fever in the physical examination. Neonates without PCT performed between H12–H36 from onset of the fever were excluded. Neonates younger than 4 days were also excluded because of high physiological PCT concentrations during the first 4 days of life (21). All clinical notes from the general pediatric department regarding neonates younger than 29 days were manually reviewed by one physician to perform the patient's selection.

### Data collection

Data retrospectively gathered from the medical records were age, sex, time from onset of fever to pediatric ED visit, CRP and PCT performed between H12-H36 from the onset of the fever, blood, urine, CSF and any other fluid cultures, duration of antibiotic therapy and final diagnoses. In addition, the neonates were categorized as either “not sick appearance” or “sick appearance” based on the medical note reported by the ED physician. “Sick appearance” was assigned if the neonate was reported to show signs of illness such as lethargy, irritability, cyanosis, dehydration or respiratory distress.

### Outcomes

Our main outcome was a diagnosis of IBI including bacteremia and bacterial meningitis, defined by the growth of a single bacterial pathogen in the blood and/or CSF. SBI were (1) IBI. (2) UTI, as a urine culture with at least 50.000 colony forming units (CFUs) per ml collected from a bag or catheterization and pyuria (defined as ≥10 white blood count (WBC)/mm3). (3) Bacterial pneumonia, as a positive pleural fluid culture result with a pathogen. (4) Bacterial gastroenteritis, as a bacterial pathogen in stool culture. (5) Bone or joint infection, as a local isolation culture of a microorganism (22).

Possible SBI was defined as a diagnosis of (1) Acute purulent otitis without bacterial documentation. (2) Non-documented mastitis. (3) Pneumonia with radiological condensation reported in the clinical notes without bacterial documentation and (4) Non-documented parotitis. Virological diagnosis was established by performing Polymerase Chain Reaction (PCR) on blood and/or CSF and/or stool and/or nasopharyngeal aspirate samples.

### Index test

Serum samples were collected during the initial visit in the ED and then between H12-H36 after the onset of fever if the ED sample were collected before H12.

PCT was defined as positive when higher to 0.6 ng/mL (23–25). We used the value of 0.6 ng/ml because some PCT results were given with a semi-quantitative dosage (< 0.6 ng/ml).

### Study size and main outcome measure

Our hypothesis was that the probability of IBI among febrile neonates with a low PCT H12-H36 would be 0. The preliminary analysis of a cohort of neonates hospitalized for acute fever in the general pediatric department over a 3-month period found a proportion of 59 % of neonates with a negative PCT H12-H36 after onset of fever (unpublished data). In order to obtain a confidence interval between 0 and 2%, it was necessary to include a minimum of 108 neonates with a low PCT assay so at least 180 febrile neonates.

### Statistical analyses

We calculated the sensitivity, specificity, positive predictive value (PPV) and negative predictive value (NPV) of PCT H12-H36 to predict IBI and SBI. These proportions are given with 95% confidence interval (95% CI). Children without available blood, urine or CSF cultures were excluded from the calculation for performance of diagnosis of IBI and SBI. We looked for those children if any diagnosis of IBI or SBI was suspected, the duration of antibiotic therapy and if they visited the ED within the following 7 days after discharge. Categorical variables were reported as absolute number/total (percentages) and compared using the Fisher exact test or χ2 test. Numerical data were shown as mean +/− standard derivation (SD) and Student's *t*-test were used. The diagnostic performances of the laboratory markers considered for detecting definite SBI and IBI were investigated by drawing a receiver operating characteristic (ROC) curve and comparing the area under the curve (AUC).

A *p*-value < 0.05 was considered statistically significant. The statistical analysis was carried out using R program for Mac (version 4.1.0). We used the STARD guidelines to report our results (26).

## Results

### Flowchart of the study and diagnosis of the patients analyzed

**Figure 1:**
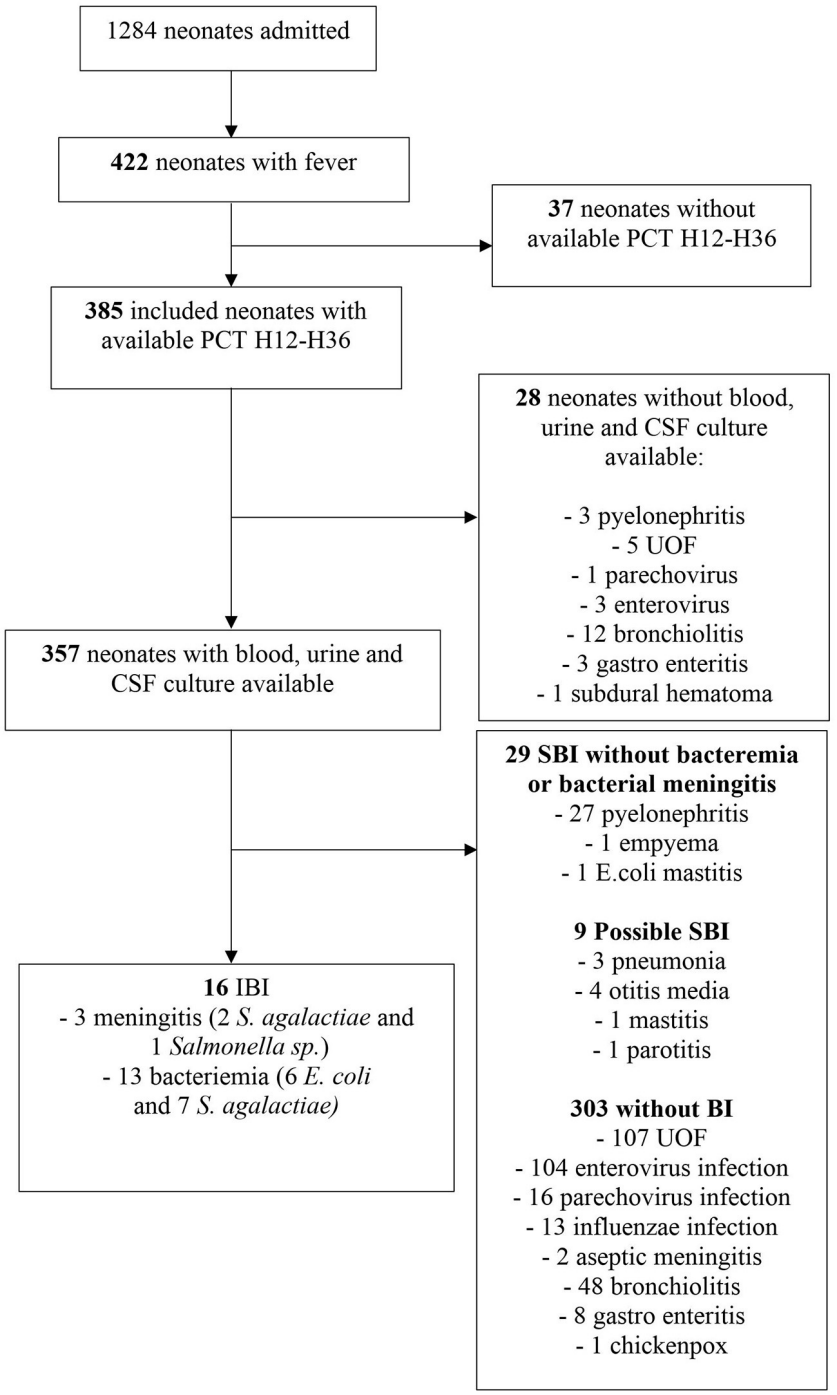
Flow chart.

Figure 1 shows the flowchart of patients through the study. Out of the 1,284 neonates admitted to the general pediatric department from January 2013 to December 2019, 422 neonates were hospitalized for acute fever. A total of 37 of them were excluded because of the absence of PCT H12-H36 assay. Among these 37 neonates, 4 had IBI (1 *Streptococcus agalactiae* meningitis and 3 *E.coli* bacteremia) and 12 had SBI (UTI). For the 4 neonates with IBI, the first PCT assay in ED performed before H12 was > 0.6 ng/ml. The 385 remaining neonates were analyzed. Blood, urine and CSF cultures were available for 357 patients (92.7%). Among the 28 neonates without available blood, urine or CSF cultures, there were no cases of IBI. The baseline demographic and clinical characteristics of the 357 included neonates are reported in Table 1. On average, they got their delayed PCT 19.5 h [± 6.8 standard deviation (SD)] after the onset of the fever. Among these 357 neonates, 45 [12.6% (95% CI: 9.3–16.5%)] had SBI: 16 IBI [4.5% [95% CI: 2.6%−7.2] with 3 meningitis (2 *Streptococcus agalactiae and* 1 *Salmonella sp.)* and 13 bacteremia (7 *S. agalactiae* and 6 *Escherichia coli)*, 27 UTI, 1 *Staphylococcus aureus* empyema, 1 *E. coli* mastitis. *E. coli* was the most common pathogen involved in 22 (81.5%) UTIs, followed by *Enterococcus faecalis* in 2 UTIs (7.4%), *Enterobacter cloacae, Staphylococcus aureus* and *S. agalactiae* with one each. The diagnoses of other patients with possible and without bacterial infection are shown in Table 1. None of the neonates with a microbiologically confirmed viral infection was also diagnosed with a concomitant bacterial infection.

**Table 1:** Clinical characteristics and diagnosis of 357 included febrile neonates.

	**All patients** **(*n* = 357)**	**Group 1** **PCT H12-H36** **≤ 0.6 ng/ml** **(*n* = 245, 68.6%)**	**Group 2** **PCT H12-H36** **> 0.6 ng/ml** **(*n* = 112, 31.4%)**	**p-value**
**Clinical characteristics**				
Number *(n, %)* Age (mean ± SD), days	357 19 ± 6.1	245 (68.8%) 19.5 ± 6	112 (31.4%) 18 ± 6.3	0.03
Sex ratio (male/female)	197/160	126/119	71/41	0.03
Clinical “sick appearing”, *(n, %)*	155 (43.4%)	92 (37.6%)	63 (56.2%)	< 0.001
Time from onset of fever to first PCT assay, (mean ± SD), hours	6.7 ± 6.3	6.8 ± 6.6	6.4 ± 5.8	0.54
Time from onset of fever to PCT H12-H36 assay, (mean ± SD), hours	19.5 ± 6.8	19.5 ± 6.6	19.5 ± 7.2	0.98
**Evolution**				
Length of stay (mean ± SD), days	3.7 ± 2.7	3.2 ± 2.1	4.9 ± 3.4	< 0.001
Duration of antibiotherapy (mean ± SD), hours	80.6 ± 117	41.5 ± 67.3	166.7 ± 152.5	< 0.001
**Diagnoses**				
SBI, *(n, %)*	45 (12.6%)	7 (2.8%)	38 (33.9%)	< 0.001
IBI *(n, %)*	16 (4.5%)	0	16 (14.3%)	< 0.001
Bacterial meningitis *(n, %)*	3 (0.8%)	0	3 (2.7%)	
Bacteremia *(n, %)*	13 (3.6%)	0	13 (11.6%)	
SBI without bacteriemia or bacterial meningitis *(n, %)*	29 (8.1%)	7 (2.8%)	22 (19.6%)	
UTI *(n, %)*	27 (7.6%)	7 (2.8%)	20 (17.8%)	
Empyema *(n, %)* Documented mastitis *(n, %)*	1 (0.3%) 1 (0.3%)	0 0	1 (0.9%) 1 (0.9%)	
Possible SBI, (n, %)	9 (2.5%)	6 (2.4%)	3 (2.7%)	0.7
Pneumonia *(n, %)*	3 (0.8%)	2 (0.8%)	1 (0.9%)	
Acute otitis media *(n, %)*	4 (1.1%)	4 (1.6%)	0	
Mastitis *(n, %)*	1 (0.3%)	0	1 (0.9%)	
Parotitis *(n, %)*	1 (0.3%)	0	1 (0.9%)	
Without BI, *(n, %)*	303 (84.9%)	232 (94.7%)	71 (63.4%)	< 0.001
UOF *(n, %)*	107 (29.9%)	87 (35.5%)	20 (17.8%)	
Enterovirus infection *(n, %)*	104 (29.1%)	65 (26.5%)	39 (34.8%)	
Parechovirus infection *(n, %)*	16 (4.5%)	8 (3.3%)	8 (7.1%)	
Influenzae virus A, B infection *(n, %)*	13 (3.6%)	12 (4.9%)	1 (0.9%)	
Aseptic meningitis *(n, %)*	2 (0.6%)	1 0.4%)	1 (0.9%)	
Bronchiolitis *(n, %)*	48 (13.4%)	46 (18.8%)	2 (1.8%)	
Gastroenteritis *(n, %)*	12 (3.4%)	12 (4.9%)	0	
Chickenpox *(n, %)*	1 (0.3%)	1 (0.4%)	0	

Percentages calculated for available data.

SD, Standard deviation; IBI, invasive bacterial infection; BI, bacterial infection; SBI, severe bacterial infection; UTI, urine tract infection; UOF, unknown origin of fever.

Regarding the 28 patients with missing urine, blood or CSF culture, 12 had respiratory syncytial virus bronchiolitis, 3 pyelonephritis (*E. coli*), 3 enterovirus infection, 1 parechovirus infection, 1 subdural hematoma, 3 gastroenteritis and 5 unknown origin fever (UOF).

### Diagnoses performances of PCT H12-H36 to predict IBI and SBI

**Figure 2:**
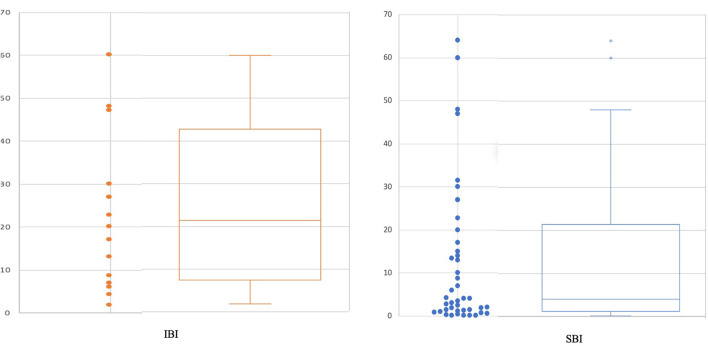
PCT H12-H36 in IBI and SBI.

**Figure 3:**
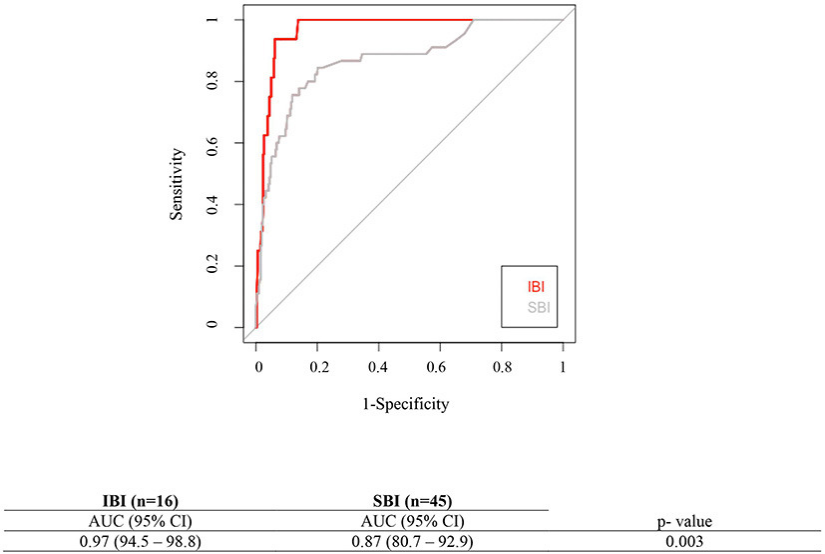
Area Under the Curve (AUC) for the Receiver Operating Characteristic Curves for PCT H12-H36 assay to detect IBI and SBI.

As showed in the Table 1, none of the 16 neonates with IBI had a negative PCT H12-H36. The performances of PCT H12-H36 assay in the identification of IBI and SBI at two selected thresholds (0.6 and 2 ng/ml) are summarized in Table 2. The median value of PCT H12-H36 for neonates with IBI was 21.3 ng/ml (range: 1.8 ng/ml−60 ng/ml). The median value of PCT H12-H36 for neonates with SBI was 3.3 ng/ml (range 0.13 g/ml and 64 ng/ml) (Figure 2). The area under the curve (AUC) receiver operating characteristic (ROC) for the detection of IBI for the PCT H12-H36 was significantly higher than that for the SBI detection (AUC, 0.95 (95% CI 94.5–98.8%); vs. AUC, 0.87 (95% CI 94.5–98.8%); *p* = 0.03) (Figure 3). Seven out of 45 neonates with SBI had a negative PCT H12-H36. The 7 SBI neonates with a negative PCT H12-H36 had UTI with leukocyturia and positive culture with a single germ (*E. coli*).

**Table 2:** Sensitivity, Specificity, positive and negative predictive values (95% CIs) for IBI and SBI at various thresholds.

**Biomarkers**	**Sensitivity**	**Specificity**	**Positive predictive values**	**Negative predictive values**
**IBI**				
PCT H12–H36 **>** 0.6 ng/ml	100 (82.9–100)	71.8 (66.8–76.6)	14.3 (8.4–22.2)	100 (98.8–100)
PCT H12–H36 **>** 2 ng/ml	93.8 (69.8–99.8)	87.4 (83.4–90.7)	25.9 (15.3–39)	99.7 (15.3–39)
**SBI**				
PCT H12–H36 **>** 0.6 ng/ml	62.2 (46.5–76.2)	90.4 (86.6–93.4)	48.3 (35–61.8)	94.3 (91.1–96.7)
PCT H12–H36 **>** 2 ng/ml	84.4 (70.5–93.5)	76.3 (71.2–80.9)	33.9 (25.3–43.5)	97.1 (94.2–98.8)

IBI, invasive bacterial infection; BI, bacterial infection; SBI, severe bacterial infection; PCT, procalcitonin.

**Figure 4:**
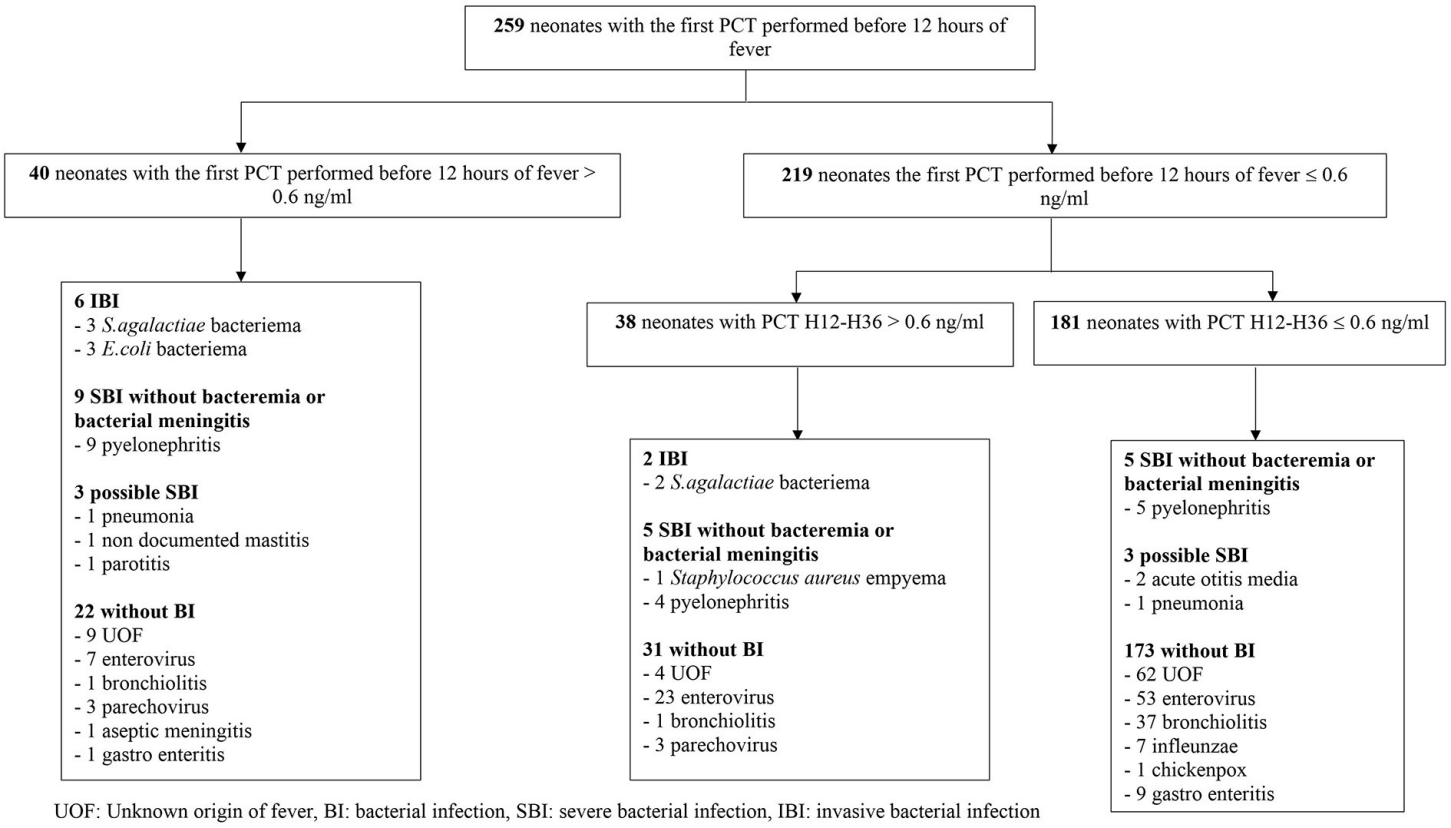
Diagnoses of neonates that had both a PCT within the first 12 h of the fever and a PCT H12-H36.

On average, neonates got their first PCT test in ED 6.7 h [± 6.1 standard deviation (SD)] after the onset of the fever and 259 patients (72.5%) had a first PCT assay within the first 12 h after the onset of the fever. Among those 259 patients with both a PCT within the first 12 h of the fever and a PCT H12-H36, 8 patients had an IBI (Figure 4 online). Two out of those 8 presented *S. agalactiae* bacteremia but had a negative initial PCT, performed respectively 1 and 3 h after onset of fever, but a positive PCT H12-H36.

## Discussion

The prevalence of SBI and IBI in our study was 12.6 and 4.5%, respectively, which is similar to what has been reported in other publications (27, 28). It is difficult to identify bacterial infections, including severe bacterial infections like SBI or IBI of febrile newborns.

During recent decades, a lot of effort has been made to identify febrile young infants at low and high risk for SBI and IBI. The different studies performed in infants < 90 days found that PCT assay performance were higher than CRP and neutrophils assays performances to identify SBI and IBI (23, 25, 29–31). Two prospective studies assessed the performance of PCT assay; *Maniaci et al*. included 234 febrile infants aged 0 to 90 days and reported an area under curve (AUC) for PCT (threshold of 0.13 ng/ml) of 0.82 for definite SBI, this value being greater than those for WBC and neutrophils (29). More recently, a french study showed that PCT had better test characteristics when compared with neutrophils and WBC for diagnosing IBIs in febrile infants aged 7 to 91 days and similar diagnostic properties as CRP for detecting SBI (23). The retrospective studies of Gomez et al. and *Olaciergi et al*. included 1,112 and 347 infants respectively and found better AUC for PCT than for CRP in identifying IBIs. Among patients with fever of recent onset, PCT remains the most accurate blood test (25, 30). Recently, *Kupperman et al*. validated a prediction rule to identify febrile infants 60 days or younger at low risk of SBI including PCT assay (PECARN rule) (18).

In our study, conducted in 357 neonates hospitalized for acute fever, no cases of IBI were missed by negative PCT H12-H36 whereas 2 IBI were missed by initial negative PCT assay performed before H12 of fever onset. These results are in agreement with a recent Spanish study that warned that the PECARN clinical rule for identification of IBI should be applied with caution in young infants with a short history of fever (32).

Furthermore, although some studies have shown a lower risk of SBI in patients with a documented viral infection, the diagnosis of a viral infection in a neonate does not rule out an associated bacterial infection. Thus, because of the high risk of SBI in this population, some authors recommend that a complete sepsis evaluation and routine antibiotic administration should be performed to all febrile neonates, including those with viral infections such a bronchiolitis (31, 33, 34). According to our data, the identification of IBI in febrile neonates can be improved using PCT H12-H36. The PCT H12-H36 is less effective for the diagnosis of SBI but SBI can be ruled out by the normality of the complete physical examination and urine test. UTIs, the most frequent SBI, accounting for 64% of all SBIs can be more easily identified with urine test results. PCT remains useful in UTIs as a predictor of both late renal scars and vesico-ureteral reflux (35).

This study is the first to evaluate the value of PCT according to its kinetics in relation to the onset of fever in neonates. The benefit would be that, after ruling out a UTI with urine test and focal bacterial infection with physical examination, a negative PCT H12-H36 in a febrile neonate might rule out an IBI and may allow antibiotic therapy to be discontinued before to have the definitive bacteriological results of the blood culture and the lumbar puncture. Several publications have assessed the use of PCT kinetics as an aid in deciding on the duration of antibiotic therapy (15–17). A recent study from the PECARN network reported that, in febrile infants ≤ 60 days of age, 82.4% (95% CI, 71.8–90.3) and 81.8% (95% CI, 59.7%−94.8%) of blood and CSF cultures showed bacterial pathogen positivity rapidly within 24 h (36). However, in practice, the turnaround time for bacteriological results is lengthened due to the absence of a laboratory within the hospital itself or the absence of a technique performed at night. Thus, in our study, we did not have definitive bacteriological results within the first 24 h for about 20% of the newborns. Currently, the difficulty in the management of febrile neonates is to find rules to stop the probabilistic antibiotic therapy started in the ED. Our study suggests that the PCT H12-H36 could be integrated into the clinical decision rules and that its negativity allows the early stopping of antibiotic treatment in the event of normal physical examination and absence of UTI.

The study has some limitations. First, we only had 16 cases of IBI, due to the relative scarcity of these infections. Second, its retrospective design and based on standard of care may affect the quality of data collection. Some patients were excluded of the PCT performance calculations because not all biological tests were performed. Follow-up information was not recorded after hospital discharge and we cannot completely rule out the possibility of initially misdiagnosed SBI. However, this possibility is unlikely; the parents would have returned to our hospital emergency room and/or the parents would have contacted the service again if the outcome had not been favorable. Third, the time of PCT determination was calculated in relation to the time of onset of fever measured and reported at home by the parents. Therefore, we may have underestimated the actual duration of fever. Fourth, there was no information in the medical record about the method of urine collection (using urine bag or urethral catheterization). Fifth, we used a cut-off value for PCT of 0.6 ng/ml which is higher than the 0.5 ng/ml cut -off commonly used in the literature. According to the report by *Van den Bruel et al*., PCT levels < 0.5 or ≥ 2 ng/ml, respectively exclude or confirm the diagnosis of SBI in febrile children (37) and recently *Kuppermann et al*. used a cut-off value for PCT > 1.71 ng/ml to predict SBI febrile infants 60 days and younger (18). In our study, the minimal PCT H12-H36 value for IBI was 1.8 ng/ml. Finally, we could not specifically test PCT at H12 of the onset of fever but on average, the patients included in our study got their delayed PCT 19.5 h after the onset of the fever.

Multicentric prospective studies are needed on a larger number of patients in order to evaluate, in these febrile newborns, whether a clinical decision rule integrating physical examination, PCT determination at H12 and urinalysis allows early discontinuation of the initially prescribed antibiotic therapy.

In conclusion, PCT H12-H36 ≤ 0.6 ng/ml seems to have good performance to rule out IBI in febrile neonates since a PCT H12-H36 ≤ 0.6 ng/ml did not miss any IBI in our study. All of the SBI cases undetected by the PCT assay were UTIs. The use of PCT between 12 and 36 h might be included in clinical decision rule to help physicians to stop early antibiotics in febrile neonates without UTIs or apparent sources.

## Data availability statement

The original contributions presented in the study are included in the article/supplementary material, further inquiries can be directed to the corresponding author.

## Ethics statement

The study was approved by the Institutional Ethics Committee of the Robert Debré Hospital (AP-HP), Paris, France (number 2018–411).

## Author contributions

A-SR, RG, EG, and ML contributed to conception and design of the study. A-SR performed the formal analysis and interpreted findings and wrote the first draft of the manuscript. All authors contributed to manuscript revision, read, and approved the submitted version.

## Conflict of interest

The authors declare that the research was conducted in the absence of any commercial or financial relationships that could be construed as a potential conflict of interest.

## Publisher's note

All claims expressed in this article are solely those of the authors and do not necessarily represent those of their affiliated organizations, or those of the publisher, the editors and the reviewers. Any product that may be evaluated in this article, or claim that may be made by its manufacturer, is not guaranteed or endorsed by the publisher.
